# Supplementary material for: Impaired Innate Immunity in Pediatric Patients Type 1 Diabetes—Focus on Toll-like Receptors Expression
Source: Int J Mol Sci. 2021 Nov 9;22(22):12135. doi: 10.3390/ijms222212135 (PMC8625857; doi:10.3390/ijms222212135)
Supplement: Supplementary file 1 [file ijms-22-12135-s001.zip › ijms-1412723-supplementary.pdf]

Supplementary Table S1. Toll-like receptors −2, −4 and −9 expression on T and B cells.

Parameter		Mean	Median	Minimum	Maximum	SD
**CD19+ TLR2+ B lymphocytes** [%]	Study group	3.84	2.45	0.17	21.22	4.26
	Group I	3.62	3.09	0.17	21.22	4.15
	Group II	4.15	2.40	0.54	19.29	4.48
	Controls	3.24	2.15	0.43	10.07	2.88
**CD19+ TLR2+ B lymphocytes** [10^3/mm^3]	Study group	0.102	0.065	0.006	0.500	0.110
	Group I	0.101	0.065	0.006	0.500	0.115
	Group II	0.104	0.066	0.010	0.500	0.110
	Controls	0.067	0.049	0.015	0.246	0.059
**CD4+ TLR2+ lymphocytes** [%]	Study group	1.83	1.21	0.12	8.49	1.85
	Group I	2.09	1.39	0.12	8.49	2.01
	Group II	1.47	0.98	0.15	6.86	1.57
	Controls	0.79	0.61	0.09	1.76	0.58
**CD4+ TLR2+ lymphocytes** [10^3/mm^3]	Study group	0.050	0.032	0.004	0.330	0.060
	Group I	0.063	0.038	0.004	0.330	0.078
	Group II	0.033	0.027	0.005	0.150	0.030
	Controls	0.017	0.015	0.002	0.038	0.012
**CD8+ TLR2+ lymphocytes** [%]	Study group	2.37	1.62	0.31	13.42	2.75
	Group I	2.05	1.55	0.31	11.79	2.18
	Group II	2.82	1.77	0.48	13.42	3.40
	Controls	2.94	2.47	0.68	6.57	1.57
**CD8+ TLR2+ lymphocytes** [10^3/mm^3]	Study group	0.060	0.039	0.003	0.310	0.060
	Group I	0.058	0.035	0.003	0.310	0.062
	Group II	0.064	0.043	0.012	0.280	0.070
	Controls	0.071	0.062	0.017	0.229	0.053
**CD19+ TLR4+ B lymphocytes** [%]	Study group	1.53	0.97	0.14	13.59	2.13
	Group I	1.42	0.67	0.16	13.59	2.55
	Group II	1.69	1.09	0.14	5.31	1.42
	Controls	0.76	0.68	0.33	1.87	0.43
**CD19+ TLR4+ B lymphocytes** [10^3/mm^3]	Study group	0.040	0.024	0.002	0.350	0.050
	Group I	0.039	0.019	0.002	0.350	0.066
	Group II	0.041	0.028	0.007	0.120	0.03
	Controls	0.018	0.015	0.004	0.047	0.013
**CD4+ TLR4+ lymphocytes** [%]	Study group	1.26	0.52	0.09	13.42	2.26
	Group I	0.80	0.51	0.09	5.83	1.10
	Group II	1.88	0.59	0.22	13.42	3.18
	Controls	0.52	0.47	0.09	1.30	0.33
**CD4+ TLR4+ lymphocytes** [10^3/mm^3]	Study group	0.031	0.015	0.001	0.280	0.050
	Group I	0.024	0.011	0.001	0.150	0.030

	Group II	0.041	0.017	0.005	0.280	0.060
	Controls	0.012	0.008	0.001	0.032	0.009
**CD8+ TLR4+ lymphocytes** [%]	Study group	1.03	0.78	0.04	6.42	1.09
	Group I	0.94	0.58	0.04	6.42	1.29
	Group II	1.16	0.95	0.17	2.72	0.76
	Controls	0.55	0.56	0.19	0.96	0.25
**CD8+ TLR4+ lymphocytes** [10^3/mm^3]	Study group	0.028	0.019	0.001	0.170	0.030
	Group I	0.028	0.014	0.001	0.170	0.036
	Group II	0.029	0.025	0.006	0.080	0.020
	Controls	0.013	0.012	0.004	0.025	0.007
**CD19+ TLR9+ B lymphocytes** [%]	Study group	4.03	2.82	0.20	18.01	3.46
	Group I	2.48	2.42	0.20	6.94	1.61
	Group II	6.22	5.63	1.03	18.01	4.16
	Controls	5.08	4.31	0.65	14.38	3.55
**CD19+ TLR9+ B lymphocytes** [10^3/mm^3]	Study group	0.110	0.080	0.004	0.590	0.100
	Group I	0.074	0.065	0.003	0.220	0.056
	Group II	0.160	0.111	0.038	0.590	0.140
	Controls	0.109	0.100	0.023	0.285	0.072
**CD4+ TLR9+ lymphocytes** [%]	Study group	1.75	0.98	0.15	11.96	2.04
	Group I	1.31	0.87	0.15	4.34	1.11
	Group II	2.35	1.72	0.20	11.96	2.81
	Controls	1.71	0.85	0.27	7.26	2.00
**CD4+ TLR9+ lymphocytes** [10^3/mm^3]	Study group	0.050	0.027	0.003	0.390	0.070
	Group I	0.039	0.026	0.003	0.120	0.036
	Group II	0.066	0.032	0.008	0.390	0.090
	Controls	0.040	0.018	0.008	0.203	0.051
**CD8+ TLR9+ lymphocytes** [%]	Study group	2.11	1.47	0.10	11.65	2.09
	Group I	1.42	1.37	0.10	3.11	0.70
	Group II	3.13	2.43	0.65	11.65	2.94
	Controls	2.44	1.98	0.31	6.89	1.78
**CD8+ TLR9+ lymphocytes** [10^3/mm^3]	Study group	0.057	0.042	0.000	0.380	0.070
	Group I	0.041	0.037	0.002	0.130	0.025
	Group II	0.078	0.050	0.000	0.380	0.100
	Controls	0.057	0.043	0.005	0.191	0.048

The level of significance of differences between the studied groups in the percentage and number of parameter.

Parameter	Study group/Controls	Group I/Controls	Group II/Controls	Group I/Group II
**CD19+ TLR2+ B lymphocytes** [%]	NS	NS	NS	NS
**CD19+ TLR2+ B lymphocytes** [10^3/mm^3]	NS	NS	NS	NS
**CD4+ TLR2+ lymphocytes** [%]	**0.018**	**0.010**	NS	NS
**CD4+ TLR2+ lymphocytes** [10^3/mm^3]	**0.003**	**0.002**	**0.040**	NS
**CD8+ TLR2+ lymphocytes** [%]	**0.026**	**0.025**	NS	NS
**CD8+ TLR2+ lymphocytes** [10^3/mm^3]	NS	NS	NS	NS

**CD19+ TLR4R+ lymphocytes** [%]	NS	NS	**0.021**	NS
**CD19+ TLR4R+ lymphocytes** [$10^3/mm^3$]	NS	NS	**0.018**	NS
**CD4+ TLR4R+ lymphocytes** [%]	NS	NS	NS	NS
**CD4+ TLR4R+ lymphocytes** [$10^3/mm^3$]	NS	NS	**0.027**	NS
**CD8+ TLR4 + lymphocytes** [%]	NS	NS	**0.006**	**0.035**
**CD8+ TLR4 + lymphocytes** [$10^3/mm^3$]	**0.040**	NS	**0.004**	NS
**CD19+ TLR9 + B lymphocytes** [%]	NS	**0.004**	NS	**0.001**
**CD19+ TLR9 + B lymphocytes** [$10^3/mm^3$]	NS	NS	NS	**0.003**
**CD4+ TLR9 + lymphocytes** [%]	NS	NS	NS	NS
**CD4+ TLR9 + lymphocytes** [$10^3/mm^3$]	NS	NS	NS	NS
**CD8+ TLR9 + lymphocytes** [%]	NS	**0.040**	NS	**0.016**
**CD8+ TLR9 + lymphocytes** [$10^3/mm^3$]	NS	NS	NS	NS

(NS—not significant).

Supplementary Table S2. Selected plasma cytokine levels.

Parameter		Mean	Median	Minimum	Maximum	SD
**IFN-γ** (pg/mL)	Study Group	4.76	4.61	2.90	8.62	1.08
	Group I	4.57	4.38	2.90	6.72	0.98
	Group II	5.06	5.00	3.08	8.62	1.18
	control group	4.58	4.64	3.00	6.71	1.06
**IL-2** (pg/mL)	Study Group	0.15	0.11	0.07	1.43	0.19
	Group I	0.13	0.12	0.07	0.39	0.07
	Group II	0.17	0.11	0.09	1.43	0.28
	control group	0.14	0.10	0.07	0.72	0.16
**IL-4** (pg/mL)	Study Group	3.34	3.21	0.90	8.60	1.68
	Group I	3.68	3.71	1.12	8.60	1.85
	Group II	2.97	2.66	0.90	5.92	1.34
	control group	3.01	3.10	1.21	6.59	1.22
**IL-10** (pg/mL)	Study Group	0.58	0.51	0.13	1.74	0.30
	Group I	0.65	0.62	0.13	1.74	0.31
	Group II	0.46	0.40	0.17	1.27	0.25
	control group	0.43	0.44	0.09	0.82	0.18

The level of significance of differences between the studied groups in the parameter concentration.

Parameter	Study Group/control group	Group I/control group	Group II/control group	Group I/Group II
**IFN-γ** (pg/mL)	NS	NS	NS	NS
**IL-2** (pg/mL)	NS	NS	**0.030**	NS
**IL-4** (pg/mL)	NS	NS	NS	NS
**IL-10** (pg/mL)	NS	**0.005**	NS	**0.003**

(NS—not significant).
